# Prenatal Exposure to Ambient Air Pollution and Epigenetic Aging at Birth in Newborns

**DOI:** 10.3389/fgene.2022.929416

**Published:** 2022-06-28

**Authors:** Ashley Y. Song, Jason I. Feinberg, Kelly M. Bakulski, Lisa A. Croen, M. Daniele Fallin, Craig J. Newschaffer, Irva Hertz-Picciotto, Rebecca J. Schmidt, Christine Ladd-Acosta, Heather E. Volk

**Affiliations:** ^1^ Department of Mental Health, Johns Hopkins Bloomberg School of Public Health, Baltimore, MD, United States; ^2^ Wendy Klag Center for Autism and Developmental Disabilities, Johns Hopkins Bloomberg School of Public Health, Baltimore, MD, United States; ^3^ Department of Epidemiology, School of Public Health, University of Michigan, Ann Arbor, MI, United States; ^4^ Division of Research, Kaiser Permanente, Oakland, CA, United States; ^5^ College of Health and Human Development, Pennsylvania State University, State College, PA, United States; ^6^ Department of Public Health Sciences, UC Davis, Davis CA and the UC Davis MIND Institute, Sacramento, CA, United States; ^7^ Department of Epidemiology, Johns Hopkins Bloomberg School of Public Health, Baltimore, MD, United States

**Keywords:** air pollution, epigenetic aging, epigenetics, prenatal exposure, DNA methylation, biologic age, ambient air pollution

## Abstract

*In utero* air pollution exposure has been associated with adverse birth outcomes, yet effects of air pollutants on regulatory mechanisms in fetal growth and critical windows of vulnerability during pregnancy are not well understood. There is evidence that epigenetic alterations may contribute to these effects. DNA methylation (DNAm) based age estimators have been developed and studied extensively with health outcomes in recent years. Growing literature suggests environmental factors, such as air pollution and smoking, can influence epigenetic aging. However, little is known about the effect of prenatal air pollution exposure on epigenetic aging. In this study, we leveraged existing data on prenatal air pollution exposure and cord blood DNAm from 332 mother-child pairs in the Early Autism Risk Longitudinal Investigation (EARLI) and Markers of Autism Risk in Babies-Learning Early Signs (MARBLES), two pregnancy cohorts enrolling women who had a previous child diagnosed with autism spectrum disorder, to assess the relationship of prenatal exposure to air pollution and epigenetic aging at birth. DNAm age was computed using existing epigenetic clock algorithms for cord blood tissue—Knight and Bohlin. Epigenetic age acceleration was defined as the residual of regressing chronological gestational age on DNAm age, accounting for cell type proportions. Multivariable linear regression models and distributed lag models (DLMs), adjusting for child sex, maternal race/ethnicity, study sites, year of birth, maternal education, were completed. In the single-pollutant analysis, we observed exposure to PM_2.5,_ PM_10,_ and O_3_ during preconception period and pregnancy period were associated with decelerated epigenetic aging at birth. For example, pregnancy average PM_10_ exposure (per 10 unit increase) was associated with epigenetic age deceleration at birth (weeks) for both Knight and Bohlin clocks (*β* = −0.62, 95% CI: −1.17, −0.06; *β* = −0.32, 95% CI: −0.63, −0.01, respectively). Weekly DLMs revealed that increasing PM_2.5_ during the first trimester and second trimester were associated with decelerated epigenetic aging and that increasing PM_10_ during the preconception period was associated with decelerated epigenetic aging, using the Bohlin clock estimate. Prenatal ambient air pollution exposure, particularly in early and mid-pregnancy, was associated with decelerated epigenetic aging at birth.

## Introduction

*In utero* exposure to air pollution is an established risk factor for low birth weight, intrauterine growth restriction, and preterm birth (Stieb et al., 2012; Lamichhane et al., 2015; Li et al., 2017). Growing evidence suggests that prenatal and early life exposure to air pollution can have profound impacts on health outcomes across the life span, yet the underlying biological mechanisms driving such associations are not well understood. Epidemiological and clinical studies have linked prenatal air pollution exposure to epigenetic alterations, inflammation, and oxidative stress (Janssen et al., 2013; Breton et al., 2016; Grevendonk et al., 2016; Saenen et al., 2016; Gruzieva et al., 2017; Breton et al., 2019). As postulated by the Developmental Origins of Health and Disease (DOHaD) hypothesis (Barker, 2007), these alterations due to air pollution during fetal programming can have long-lasting effects on biological function, which in turn can influence susceptibility to diseases in later life.

DNA methylation (DNAm), the most studied epigenetic mechanism with a crucial role in maintaining genomic stability and regulation of gene function, has been observed to be altered in association with environmental exposures (Baccarelli and Bollati, 2009; Martin and Fry, 2018). Epigenome-wide association studies (EWAS) have investigated both short-term and long-term exposure to ambient and traffic-related air pollutants in children and adult populations (Isaevska et al., 2021; Wu et al., 2021). Studies have reported the effects of prenatal exposure to air pollutants on DNAm changes at the global, region-specific, and site-specific level (Breton et al., 2016; Gruzieva et al., 2017; Neven et al., 2018; Gruzieva et al., 2019; Ladd-Acosta et al., 2019).

More recently, DNAm based or epigenetic age estimates have emerged as a promising biomarker of biological aging across tissues and ethnicities (Horvath, 2013; Horvath et al., 2016; Horvath and Raj, 2018), as compared to other molecular biomarkers including telomere length or age estimation based on the transcriptome. The difference between an individual’s chronologic age and predicted epigenetic age captures the age discordance of the individual. Epigenetic age acceleration, having an estimated epigenetic age that exceeds actual chronological age, has been studied in association with health outcomes and age-related conditions (Marioni et al., 2015; Chen et al., 2016; Perna et al., 2016; Levine et al., 2018). Emerging evidence suggests that epigenetic age acceleration or deceleration can be moderated by lifestyle and environmental factors such as cigarette smoking, socioeconomic status, body mass index, and air pollution (Horvath et al., 2014; Quach et al., 2017; Simpkin et al., 2017; Lu et al., 2019; Ward-Caviness et al., 2020; de Prado-Bert et al., 2021). A recent study by de Pardo-Bert et al. investigated the association between more than 100 exposures and epigenetic age acceleration during childhood using an exposome-wide approach; but no significant associations were observed between prenatal air pollution and epigenetic aging in childhood (de Prado-Bert et al., 2021). Epigenetic clocks for gestational age at birth (Bohlin et al., 2016; Knight et al., 2016; Lee et al., 2019), estimated using cord blood and placenta DNAm data, were developed to reflect the fetal developmental age and to capture the fetal programming progress. Recent studies have shown that epigenetic age acceleration or deceleration at birth is associated with maternal lifestyle, pregnancy complications, and psychosocial factors (Girchenko et al., 2017; Suarez et al., 2018; McKenna et al., 2021; Workalemahu et al., 2021). Investigation of prenatal environment and fetal aging process is still in its infancy, while most of studies have focused on other types of molecular biomarkers of aging. To our knowledge, no studies have evaluated the effect of prenatal air pollution exposure on epigenetic age acceleration at birth.

The purposes of this study were 1) to test the associations between prenatal exposure to ambient air pollution during the preconception and pregnancy periods and epigenetic age acceleration/deceleration at birth using cord blood DNAm, and 2) to identify critical windows of air pollution exposure related to epigenetic age acceleration/deceleration.

## Methods

### Study Populations

This analysis draws on two pregnancy cohorts with increased likelihood of autism spectrum disorder (ASD) and other neurodevelopmental outcomes based on prospectively following pregnant mothers, and children from that pregnancy, who have had a previous child with ASD given the high sibling recurrence risk for these conditions. The Early Autism Risk Longitudinal Investigation (EARLI) (Newschaffer et al., 2012) was implemented at four major metropolitan locations across the U.S. (Philadelphia, Baltimore, San Francisco Bay Area, and Sacramento), representing three distinct US regions (Southeast Pennsylvania, Northeast Maryland, and Northern California). Recruitment methods varied by location to capitalize on unique resources at each study site. Enrolled mothers were seen at regular intervals during pregnancy (approximately once a trimester) and at birth to complete interviews that cover a wide range of exposure, medical, and demographic domains, as well as to collect biologic and environmental samples, including cord blood and placenta at birth.

The Markers of Autism Risk in Babies, Learning Early Signs (MARBLES) study uses a similar study design but recruits Northern California mothers, pregnant or planning a pregnancy, who have a child with ASD recorded as receiving services through the California Department of Developmental Services. MARBLES requires that the mother or father has at least one biological child with ASD, the mother is at least 18 years old, that the mother speaks, reads, and understands English at a sufficient level to complete the protocol and that the younger sibling will be taught to speak English, and that the mother lives within 2.5 h of the Davis/Sacramento region at the time of enrollment. As described in detail elsewhere (Hertz-Picciotto et al., 2018), demographic, diet, lifestyle, environmental, and medical information were prospectively collected through telephone-assisted interviews and mailed questionnaires throughout pregnancy and the postnatal period. The institutional review boards (IRB) at organizations in each study site approved the EARLI and the MARBLES studies.

### Air Pollution Exposure Assignments

For both EARLI and MARBLES studies, air pollution exposure assignments were based on maternal residences recorded prospectively 3 months prior to conception and throughout pregnancy for both studies. All residential locations for each mother and child were standardized and geo-coded using the TeleAtlas US_Geo_2 database and software (Tele Atlas, Inc., Boston, CA, www.geocoded.com). Air quality assignments for particulate matter less than 2.5 and 10 microns in diameter (PM_10_, PM_2.5_), ozone (O_3_), and Nitrogen Dioxide (NO_2_), were derived from the US EPA’s Air Quality System (AQS) data (www.epa.gov/ttn/airs/airsaqs). The weekly air quality data from monitoring stations located within 50 km of each residence were made available for spatial interpolation of ambient concentrations. The spatial interpolations were based on inverse distance-squared weighting (IDW2) of data from up to four closest stations located within 50 km of each participant residence; however, if one or more stations were located within 5 km of a residence then only data from the stations within 5 km were used for the interpolation. Based on estimates of gestational age from medical record review and dates of reported residence we calculated weekly pregnancy exposures. Exposure periods were calculated based on the gestational age of the infant at birth and were divided into preconception (3 months before pregnancy), first trimester (day 1 to day 90 of pregnancy), second trimester (day 91 to day 180 of pregnancy), third trimester (day 181 of pregnancy to birth), and pregnancy (conception to birth).

### DNA Methylation Measurements and Quality Control

In both studies, umbilical cord blood biosamples were collected shortly after delivery using standardized protocol across all sites. The biosamples were shipped on the same day to the central labs for storage at −80°C. Genomic DNA was extracted using a Qiagen DNA Midi Kit (Qiagen Inc., Valencia, CA) and quantified using a NanoDrop spectrophotometer (ThermoFisher Scientific). DNA methylation was measured using the Illumina Infinium HumanMethylation450 BeadChip (EARLI) and Illumina Infinium HumanMethylationEPIC BeadChip (MARBLES) (Illumina, San Diego, CA). For each sample, 1 μg of genomic DNA was bisulfite treated using the EZ-96 DNA Methylation kit (Zymo Irvine, CA), as per the manufacturer’s instructions.

In both studies, several sample- and probe-level quality control measures were applied, as described previously (Bakulski et al., 2021; Dou et al., 2022). Samples were excluded if they were duplicates, had low overall array intensity, or a discrepancy between reported sex and empirically predicted sex. Cross-reactive probes as well as probes that measured DNA methylation at known SNP positions and outside of CpG sites were removed. Probes with detection *p*-values > 0.01 in 10% of samples for EARLI and probes with detection *p*-values>0.01 in 5% of samples for MARBLES were removed from the analyses. DNAm data were then normalized using a modified beta-mixture quantile (BMIQ) function (Horvath, 2013; Teschendorff et al., 2013) for the EARLI and the MARBLES studies separately. While the original BMIQ is a within-sample normalization method to address probe type bias by modifying the type II distribution to match that of type I probes (Teschendorff et al., 2013), Horvath modified this BMIQ procedure for a different purpose: the distribution of each given array is related to that of a “gold standard” array (defined here as the mean across all the training datasets) (Horvath, 2013). Thus, Horvath’s modification of the BMIQ method could be interpreted as a form of between sample normalization. Lastly, proportions of cell types, including B cells, CD4^+^ T cells, CD8^+^ T cells, monocytes, granulocytes, nucleated red blood cells, and natural killer cells, were empirically estimated using the estimateCellCounts function of the *minfi* R package (Houseman et al., 2012; Aryee et al., 2014). A total of 140 cord blood DNAm samples from EARLI and 192 cord blood DNAm samples from MARBLES that passed the QC procedures were included in this analysis.

### Epigenetic Age Estimation

Epigenetic age at birth was calculated using two existing epigenetic clock algorithms for cord blood DNAm samples—Knight and Bohlin (Bohlin et al., 2016; Knight et al., 2016). Both the Knight method and the Bohlin method used an elastic net approach with 10-fold cross-validation in the training set. The Knight method is based on 148 CpG sites, while the Bohlin method prediction uses 96 sites. The Knight clock and Bohlin clock were performed using R statistical software with code supplied from Knight et al. and *GAprediction* R package (Bohlin et al., 2016; Knight et al., 2016). For both EARLI and MARBLES data, a number of required CpGs for clock computation were missing due to QC filters (*n* = 7 for EARLI and *n* = 9 for MARBLES) and imputed using simple random sampling imputation. Epigenetic age acceleration is defined as the residual from a linear regression of epigenetic age on gestational age, adjusting for cell-type proportions.

### Covariate Information

Maternal, paternal, and child characteristics, including maternal age (years), race (White, Black, Asian, Other), ethnicity (Non-Hispanic, Hispanic), maternal education (high school, college, graduate school or higher), annual family income (less than $50,000, $50,001-$100,000, more than $100,0001), maternal pre-pregnancy body mass index (BMI, underweight, normal weight, overweight, obese), and child’s sex (male, female) were obtained primarily through maternal-report questionnaires at enrollment. In MARBLES, if covariate information such as maternal education and maternal BMI were not available through maternal reported questionnaires, medical records and delivery data were used for those covariates. Annual family income information was not available in the MARBLES study. Labor and delivery information, including gestational age, birth weight, and parity, were extracted from medical records by abstractors or physicians at each site.

### Statistical Analyses

Data from the EARLI and the MARBLES studies were pooled together for statistical analyses. Descriptive analyses were conducted to examine maternal, paternal, and child characteristics by study. Continuous covariates (gestational age, maternal age, birthweight, air pollution levels, cell composition) were described using mean and standard deviation and categorical covariates (maternal race, maternal ethnicity, maternal education, maternal pre-pregnancy BMI, child’s sex) were described using sample number and frequency. Spearman correlation coefficients between chronological age, estimated epigenetic age, and epigenetic age acceleration/deceleration across Knight and Bohlin clocks were calculated. Distribution of the prenatal air pollutants were examined by region and by birth year using two-tailed t-tests. Pearson correlations were used to evaluate the pairwise relationships between prenatal air pollution exposures over time. For each pollutant and each clock, separate multivariable linear regression models were completed to evaluate the association between prenatal exposure to ambient air pollution (continuous variable) and epigenetic age acceleration (continuous variable) at birth for each study adjusted for potential confounders. In addition, models mutually adjusted for different exposure periods were assessed to account for correlated time periods of exposures for each pollutant. Vulnerable windows of exposure were further investigated using weekly distributed lag models (DLMs), which accounted for both current and past values of the exposure. Natural cubic spline DLMs with 4 degrees of freedom (R package *dlnm*) were used to fit all weekly air pollution estimates from preconception period to birth (week 1–52) into one model. In sensitivity analyses, polynomial DLMs and natural spline DLMs with various degrees of freedom were also considered and compared using the Akaike information criteria (AIC). All models were adjusted for potential confounders, including region of the participants at recruitment (East Coast, West Coast), child’s sex, maternal race/ethnicity, maternal education, and year of birth. Potential confounders were selected based on prior knowledge and retained in the adjusted model if a 15% change in coefficient estimate was observed. Results are presented as *β* estimates (weeks) with 95% confidence intervals (CI) per a 10 unit increase in each exposure. A positive *β* coefficient indicates accelerated epigenetic aging or faster epigenetic aging as compared to gestational age whereas a negative *β* coefficient indicates decelerated epigenetic aging or slower epigenetic aging as compared to gestational age. A two-sided *p*-value less than 0.05 was considered statistically significant. All statistical analyses were performed using R 3.6 software. Data are available through the National Institute of Mental Health Data Archive (NDA) under the collections for the EARLI study (1600) and for the MARBLES study (1946).

## Results

The characteristics of the full population by study are presented in Table 1. Overall, mothers were predominantly White (71.6%), non-Hispanic (80.7%), and on average 34 years old at time of birth. Similar characteristics were observed in the EARLI and MARBLES study populations. The mean (SD) gestational age at birth for EARLI and MARBLES were 39.3 (1.3) weeks and 39.0 (1.3) weeks, respectively. Average (SD) exposure levels during pregnancy period for the entire study population were 11.1 (3.0) ppb for NO_2_, 25.7 (4.3) ppb for O_3_, 9.8 (2.0) μg/m^3^ for PM_2.5_, and 18.8 (3.8) μg/m^3^ for PM_10_ (Table 1, Supplementary Figure S1). We observed significantly higher levels of prenatal exposure to NO_2_ during pregnancy among those residing on the East Coast compared to those from the West Coast (*p < 0.01*, Supplementary Figure S2). Significant differences for the other pollutants during pregnancy by region were also observed (*p < 0.05 for all*) (Supplementary Figure S2). Pearson correlations between PM_10_, PM_2.5_, and NO_2_ were low to moderate (*r* range from −0.06–0.57). Preconception average O_3_ was inversely correlated with NO_2_ and PM_2.5_ (*r* = − 0.56 and −0.37, respectively) (Supplementary Figure S3).

**TABLE 1:** Descriptive statistics for our analytic sample from the Early Autism Risk Longitudinal Investigation (EARLI) and the Markers of Autism Risk in Babies, Learning Early Signs (MARBLES) studies.

Characteristic	Overall (*n* = 332)	EARLI (*n* = 140)	MARBLES (*n* = 192)
Gestational Age (weeks), mean (SD)	39.1 (1.3)	39.3 (1.3)	39.0 (1.3)
Birthweight (g), mean (SD)	3469.0 (482.0)	3467.0 (512.7)	3471.0 (459.9)
Male	192 (57.8)	76 (54.3)	116 (60.4)
Maternal age (years), mean (SD)	33.5 (4.8)	33.5 (4.7)	33.5 (4.9)
Maternal race			
White	235 (71.6)	87 (64.0)	148 (77.1)
Black	23 (7.0)	16 (11.8)	7 (3.6)
Asian	44 (13.4)	18 (13.2)	26 (13.5)
Other	26 (8.0)	15 (11.0)	11 (5.8)
Maternal ethnicity			
Non-Hispanic	268 (80.7)	120 (85.7)	148 (77.1)
Hispanic	64 (19.3)	20 (14.3)	44 (22.9)
Maternal education			
High school	33 (10.0)	15 (10.9)	18 (9.4)
College	226 (68.7)	89 (64.5)	137 (71.7)
Graduate school or higher	70 (21.3)	34 (24.6)	36 (18.9)
Maternal pre-pregnancy BMI			
Underweight	5 (1.5)	2 (1.5)	3 (1.6)
Normal weight	129 (39.5)	50 (37.0)	79 (41.1)
Overweight	99 (30.3)	40 (29.6)	59 (30.7)
Obese	94 (28.7)	43 (31.9)	51 (26.6)
Year of birth			
2006–2009	63 (19.0)	6 (4.3)	57 (29.7)
2010–2012	188 (56.6)	133 (95.0)	55 (28.6)
2013–2015	81 (24.4)	1 (0.7)	80 (41.7)
Region			
East	71 (21.4)	71 (50.7)	0 (0)
West	261 (78.6)	69 (49.3)	192 (100.0)
Cell composition, mean (SD)			
B cell	10.3 (3.7)	11.0 (3.9)	9.6 (3.5)
CD4^+^ T cell	18.9 (7.8)	19.4 (8.3)	18.5 (7.3)
CD8^+^ T cell	12.1 (4.1)	13.3 (4.2)	11.0 (3.8)
Granulocyte	44.1 (11.7)	42.8 (12.5)	45.2 (10.8)
Monocyte	8.8 (2.7)	8.4 (2.5)	9.3 (2.8)
Natural killer cell	0.5 (1.2)	0.5 (1.1)	0.5 (1.3)
Nucleated red blood cells	9.5 (5.3)	10.0 (5.3)	8.9 (5.2)
Prenatal exposure levels, mean (SD)			
NO_2_ (ppb)	11.1 (3.0)	12.2 (3.3)	10.2 (2.5)
O_3_ (ppb)	25.7 (4.3)	26.0 (4.1)	25.5 (4.4)
PM_2.5_ (μg/m^3^)	9.8 (2.0)	9.8 (1.7)	9.9 (2.2)
PM_10_ (μg/m^3^)	18.8 (3.8)	17.7 (3.0)	19.6 (4.2)
Epigenetic age by Knight (weeks), mean (SD)	37.4 (1.8)	38.0 (2.0)	37.0 (1.6)
Epigenetic age by Bohlin (weeks), mean (SD)	39.1 (1.1)	39.2 (1.1)	39.0 (1.1)

Missing data: birthweight: *n* = 1 (EARLI); maternal age: *n* = 1 (MARBLES); maternal race: *n* = 4 (EARLI); maternal education: *n* = 2 (EARLI), *n* = 1 (MARBLES); maternal BMI: *n* = 5 (EARLI). Values are number and frequency, unless otherwise stated.

DNAm estimates of epigenetic age, using the Knight and Bohlin clock algorithms for cord blood samples, showed significant high overall correlations with chronologic gestational age (Supplementary Figure S4, correlation = 0.42 and 0.52, respectively, *p* < 0.001). After residualization, epigenetic age acceleration was not associated with chronological gestational age (all *p* > 0.05).

As shown in Table 2, average O_3_ exposure during the preconception period was associated with epigenetic age deceleration at birth (per 10-unit increment, *β* = −0.35, 95% CI: −0.63, −0.07) and average O_3_ exposure during the pregnancy period was marginally associated with epigenetic age deceleration at birth (per 10-unit increment, *β* = −0.48, 95% CI: −0.96, 0.01) using the Knight clock. The Bohlin clock estimates showed a similar direction and attenuated magnitude of effect estimates but did not reach statistical significance. Effect estimates for PM_2.5_ during pregnancy and associations for both clocks suggest decelerated epigenetic aging as well, though only associations with the Bohlin clock reached statistical significance (Table 2). Marginally significant associations were observed between preconception average PM_10_ and decelerated epigenetic aging at birth. Pregnancy average PM_10_ was associated with decreased epigenetic age for both Knight and Bohlin clocks (per 10-unit increment, *β* = −0.62, 95% CI: −1.17, −0.06; *β* = −0.32, 95% CI: −0.63, −0.01, respectively) (Table 2). We did not observe any significant associations between exposure to NO_2_ during preconception and pregnancy period and epigenetic aging at birth.

**TABLE 2:** Association of pregnancy average ambient air pollution and epigenetic aging at birth in the EARLI and MARBLES cohorts.

	N	Individual Models		Mutually Adjusted Models	
Pollutant		Knight Clock	Bohlin Clock	Knight Clock	Bohlin Clock
		β (95% CI)	β (95% CI)	β (95% CI)	β (95% CI)
Preconception					
NO_2_	315	0.05 (−0.50, 0.59)	−0.05 (−0.35, 0.26)	0.006 (−0.56, 0.57)	−0.15 (−0.46, 0.17)
O_3_	313	−0.35 (−0.63, −0.07)	−0.03 (−0.19, 0.13)	−0.34 (−0.62, −0.05)	0.009 (−0.15, 0.17)
PM_2.5_	316	0.29 (−0.30, 0.88)	−0.15 (−0.48, 0.18)	0.22 (−0.40, 0.84)	−0.25 (−0.59, 0.09)
PM_10_	316	−0.38 (−0.78, 0.01)	−0.24 (−0.46, −0.01)	−0.26 (−0.68, 0.16)	−0.16 (−0.39, 0.08)
Pregnancy					
NO_2_	308	−0.16 (−0.97, 0.65)	0.11 (−0.34, 0.56)	−0.22 (−1.07, 0.63)	0.19 (−0.28, 0.67)
O_3_	304	−0.48 (−0.96, 0.01)	−0.14 (−0.42, 0.14)	−0.58 (−1.10, −0.06)	−0.21 (−0.50, 0.09)
PM_2.5_	309	−0.28 (−1.32, 0.76)	−0.67 (−1.24, −0.09)	−0.27 (−1.34, 0.80)	−0.66 (−1.25, −0.07)
PM_10_	309	−0.62 (−1.17, −0.06)	−0.32 (−0.63, −0.01)	−0.59 (−1.17, 0.001)	−0.26 (−0.59, 0.07)

The individual models were separate models for each air pollutant of each exposure period. The mutually adjusted models were one model for each air pollutant with mutually adjusted for both pregnancy and preconception period. All models were adjusted for child sex, maternal race/ethnicity, maternal education, year of birth, and region of the participant at recruitment. N represents the sample size of the air pollution estimates for each exposure period. The *β* coefficient represents the difference in epigenetic age acceleration in gestational weeks for a 10-unit difference in the pollutant. Epigenetic age acceleration was defined as the residual of epigenetic age estimated by Knight clock or Bohlin clock on gestational age at birth adjusted for cell heterogeneity.

Mutual adjustment for both preconception and pregnancy periods to account for correlation between the periods resulted in higher variance of estimates, but it revealed a stronger negative association of O_3_ with epigenetic aging by Knight clock for the pregnancy period (per 10-unit increment, *β* = −0.58, 95% CI: −1.10, −0.06) (Table 2). Associations between PM_2.5_ and PM_10_ and epigenetic aging for both exposure periods were in general attenuated in the mutually adjusted models (Table 2).

We also tested for the trimester-specific exposures and epigenetic aging associations (Table 3) in individual models and models mutually adjusted for all 3 trimester exposure periods. Trimester-specific exposures to NO_2_ and O_3_ were not significantly associated with epigenetic age acceleration/deceleration at birth for both Knight and Bohlin clock. Exposure to PM_2.5_ during first trimester was associated with epigenetic age deceleration at birth (*β* = −0.35, 95% CI: −0.69, −0.01) using the Bohlin clock. In addition, we observed epigenetic age deceleration with increases in PM_10_ during first trimester and second trimester (*β* = −0.44, 95% CI: −0.81, −0.08; *β* = −0.65, 95% CI: −0.50, −0.38, respectively) using the Knight clock (Table 3). Results were similar when mutually adjusting for all 3 trimester exposure periods with higher variance of estimates; we did observe a negative association between O_3_ during trimester 1 and epigenetic aging in the mutually adjusted models.

**TABLE 3:** Association of trimester-specific and preconception average ambient air pollution and epigenetic aging at birth.

		Individual Models		Mutually Adjusted Models	
Pollutant	N	Knight Clock	Bohlin Clock	Knight Clock	Bohlin Clock
		β (95%CI)	β (95%CI)	β (95%CI)	β (95%CI)
Trimester 1					
NO_2_	316	0.09 (−0.42, 0.60)	−0.06 (−0.35, 0.23)	0.13 (−0.49, 0.75)	−0.07 (−0.42, 0.27)
O_3_	315	−0.25 (−0.51, 0.02)	0.02 (−0.13, 0.18)	−0.39 (−0.72, −0.05)	0.05 (−0.14, 0.24)
PM_2.5_	317	−0.05 (−0.65, 0.55)	−0.35 (−0.69, −0.01)	−0.01 (−0.63, 0.61)	−0.29 (−0.64, 0.05)
PM_10_	316	−0.44 (−0.81, −0.08)	−0.15 (−0.36, 0.05)	−0.14 (−0.59, 0.30)	0.05 (−0.20, 0.30)
Trimester 2					
NO_2_	318	−0.25 (−0.79, 0.29)	0.10 (−0.20, 0.40)	−0.14 (−0.80, 0.51)	0.14 (−0.22, 0.51)
O_3_	316	−0.12 (−0.40, 0.15)	−0.10 (−0.25, 0.05)	−0.10 (−0.38, 0.19)	−0.12 (−0.28, 0.04)
PM_2.5_	319	−0.17 (−0.75, 0.40)	−0.13 (−0.45, 0.18)	−0.24 (−0.82, 0.34)	−0.17 (−0.49, 0.15)
PM_10_	318	−0.65 (−1.04, −0.25)	−0.27 (−0.49, -0.05)	−0.67 (−1.17, −0.17)	−0.25 (−0.53, 0.03)
Trimester 3					
NO_2_	315	−0.16 (−0.64, 0.31)	0.04 (−0.23, 0.31)	−0.13 (−0.71, 0.44)	−0.02 (−0.34, 0.30)
O_3_	315	−0.03 (−0.29, 0.22)	−0.015 (−0.16, 0.13)	−0.18 (−0.50, 0.13)	0.06 (−0.12, 0.23)
PM_2.5_	316	−0.28 (−0.83, 0.27)	−0.18 (−0.49, 0.13)	−0.41 (−0.97, 0.16)	−0.28 (−0.59, 0.03)
PM_10_	315	−0.06 (−0.50, 0.38)	−0.18 (−0.43, 0.06)	0.09 (−0.39, 0.58)	−0.11 (−0.38, 0.17)

The individual models were separate models for each air pollutant of each exposure period. The mutually adjusted models were one model for each air pollutant with mutually adjusted for trimester 1, trimester 2, and trimester 3 period. N represents the sample size of the individual models for each exposure period. The *β* coefficient represents the difference in epigenetic age acceleration for a 10-unit difference in the pollutant. Epigenetic age acceleration was defined as the residual of epigenetic age estimated by Knight clock or Bohlin clock on gestational age at birth adjusted for cell heterogeneity.

**FIGURE 1:**
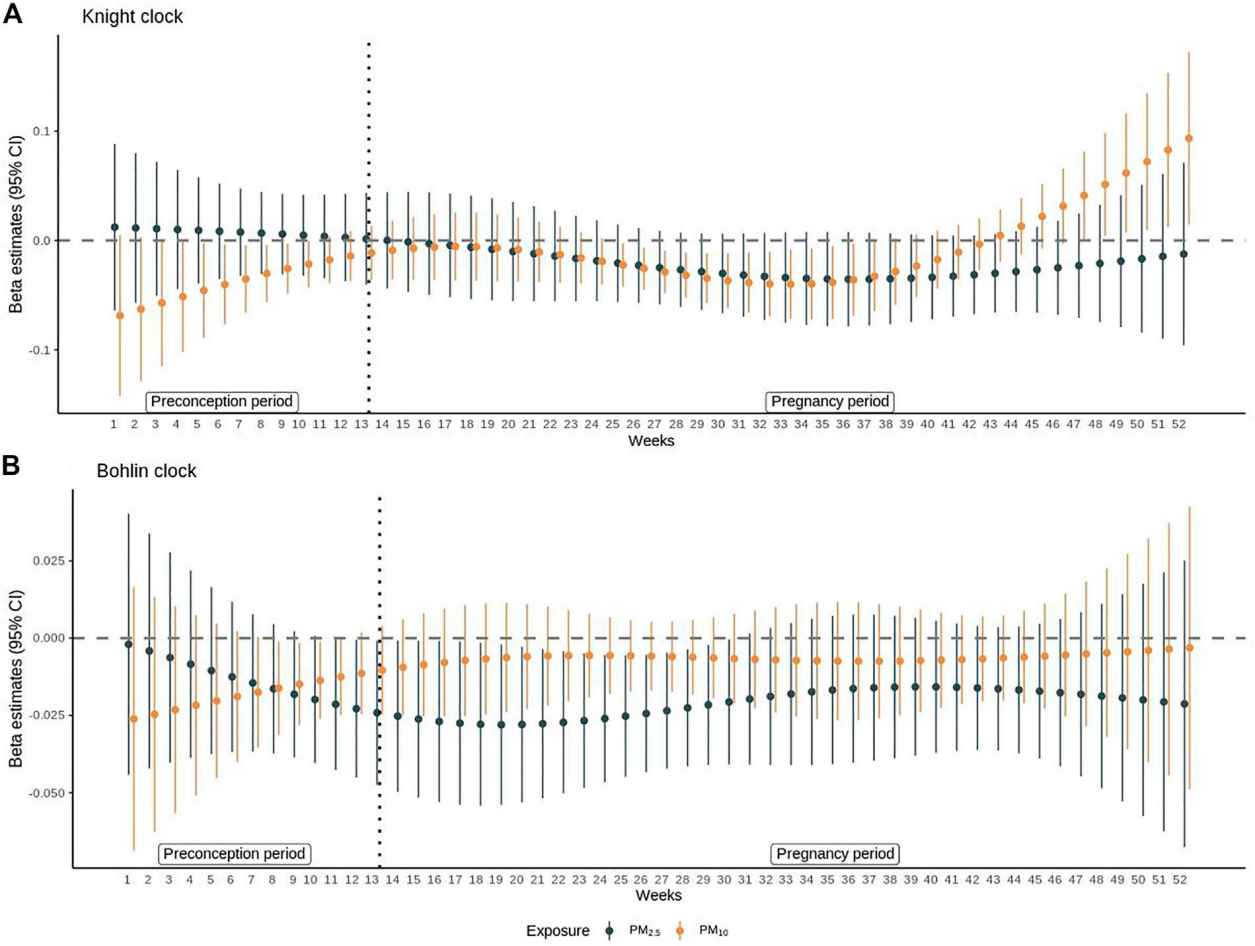
Distributed lag model results for PM_2.5_ and PM_10**.**_ The *β* coefficients and 95% confidence intervals from distributed lag models (DLMs) are shown for associations between **(A)** PM_2.5_ or PM_10_ and epigenetic age acceleration estimated by Knight et al. **(B)** PM_2.5_ or PM_10_ and epigenetic age acceleration estimated by Bohlin et al. at each week of preconception (week 1–13) and pregnancy (week 14–52). All models were adjusted for child sex, maternal race/ethnicity, maternal education, year of birth, and region of the participant at recruitment. The *β* coefficient represents the difference in epigenetic age acceleration for a 10-unit difference in the pollutant. Epigenetic age acceleration was defined as the residual of epigenetic age estimated by Knight clock or Bohlin clock on gestational age at birth adjusted for cell heterogeneity.

Results of DLMs for PM_2.5_ and PM_10_ are shown in Figure 1 and are shown for other pollutants in Supplementary Figures S5, S6. Exposure to PM_2.5_ during weeks 12–29 of the entire preconception and pregnancy period was significantly associated with epigenetic age deceleration using the Bohlin clock estimates. PM_10_ exposure was significantly associated with epigenetic age deceleration between weeks 6–11 of preconception period and weeks 13–24 of pregnancy period (weeks 26–37 of the entire preconception and pregnancy period) using the Knight clock (*p* < 0.05 for all). The strongest association between PM_2.5_ and epigenetic age deceleration was observed for exposure at week 6 of pregnancy (week 19 of entire preconception and pregnancy period) (*β*, −0.03, 95% CI: −0.05, −0.02), and the strongest association between PM10 and epigenetic age deceleration was observed for exposure at week 4 of preconception (*β* = −0.05, 95% CI: −0.10, −0.09). DLMs did not show any statistically significant association between NO_2_ and epigenetic aging at birth in both clock estimates. Exposure to O_3_ during week 6 to week 13 of preconception was associated with epigenetic age deceleration using the Knight clock (Supplementary Figure S6); similar associations were not observed using the Bohlin clock.

## Discussion

In this study, we observed that exposure to PM_2.5_ during pregnancy was associated with decelerated epigenetic aging and that exposure to PM_10_ during the preconception period, second trimester, and the overall pregnancy period were associated with decelerated epigenetic aging. Exposure to O_3_ during the preconception period, first trimester, and the pregnancy average was also associated with epigenetic age deceleration. Nitrogen dioxide was not significantly associated with epigenetic aging at birth. The DLMs revealed that an inverse association between PM_2.5_ and epigenetic age deceleration during weeks 12–29 of the preconception and pregnancy periods. In contrast, PM_10_ exposure throughout preconception and during weeks 13–24 of pregnancy period was associated with decelerated epigenetic age at birth.

The effects of prenatal air pollution on preterm birth and low birth weight are well documented (Stieb et al., 2012; Lamichhane et al., 2015). However, few studies have examined the biological aging process in tissues relevant to fetal programming. Most of those studies have focused on the molecular biomarkers of biological aging such as telomere length in response to prenatal air pollution (Martens et al., 2017; Isaevska et al., 2021). Martens et al., reported that higher levels of PM_2.5_ during second trimester were associated with shorter telomere lengths in cord blood and placenta in the *ENVIRONAGE* birth cohort (Martens et al., 2017). Our study observed epigenetic age deceleration at birth related to exposure to PM during pregnancy. We also identified that early to mid-pregnancy is a critical window for the association between PM and epigenetic aging, which is consistent with Martens et al. To our knowledge, this is the first study to investigate such an association using the epigenetic age estimators from cord blood DNAm. Our findings contribute to the growing body of evidence that prenatal environmental stress may influence the fetal programming at molecular level captured by epigenetic aging, which may be a biomarker of fetal maturity through pregnancy. Understanding the fetal aging process and mechanism which influence this process requires additional investigation.

A recent review of epidemiological studies and animal models indicate that exposures occurring both prior to conception and at the time of conception can shape fetal growth and thereby influence eventual pregnancy and birth outcomes (Fleming et al., 2018). For example, there is evidence that supports a potential role for preconception exposure air pollution (3 months prior to conception) in association with neurodevelopment and respiratory health in children (Kuiper et al., 2020; McGuinn et al., 2020). In the current study, we identified that exposure to PM_10_ during the 6–11 weeks prior to conception was significantly inversely associated with epigenetic aging at birth. Exposure to PM during preconception has been linked to gestational diabetes mellitus, inflammation during pregnancy, and fetal growth (Robledo et al., 2015; Nachman et al., 2016; Najafi et al., 2020), suggesting that preconception exposures may affect the growth and development of the placenta and the fetus. Findings from a Chinese study using weekly DLMs showed that exposure to PM_2.5_ during the 1–9 weeks prior to conception was associated with fetal undergrowth (Chen et al., 2022). Taken together, the evidence from literature and the observed association in our study demonstrates the preconception period as an emerging exposure period of interest for future research on the developing fetus and subsequent health outcomes.

A recent exposome-wide association study highlighted the association between early life environmental exposures and epigenetic age acceleration or deceleration in children utilizing the Horvath Skin and Blood clock (de Prado-Bert et al., 2021). Several cohort studies in adult populations have reported exposure to ambient PM associated with epigenetic aging using the Horvath, Hannum and Levine epigenetic clocks (Nwanaji-Enwerem et al., 2016; Ward-Caviness et al., 2016; White et al., 2019; Ward-Caviness et al., 2020). The utility of epigenetic clocks as a promising molecular biomarker to investigate the biological aging and its relationship to environmental exposures and adverse health outcomes is growing; however, the investigation of the epigenetic clocks during the early life is underrepresented. Epigenetic clocks in tissues relevant to early life exposures and epigenetic clocks enriched for clinical and biological factors have since been developed to accurately capture the biological processes not represented by Horvath, Hannum and Levine clocks. Our study evaluates the effects of air pollution on epigenetic clocks developed specifically using cord blood DNAm at birth. Estimates from the single-pollutant model analyses showed similar direction of effect for both Knight and Bohlin clocks for most exposure periods. However, results from the DLMs were inconsistent for the two clocks when examined in relation to the same air pollutant. The lack of correspondence across the two clocks in the DLMs may indicate the differences between the clocks potentially capturing unique fetal maturity process during specific gestational period as evidenced by their imperfect correlations with each other and gestational age.

To the best of our knowledge, this is the first study to evaluate whether preconception and prenatal exposure to air pollution is associated with epigenetic aging at birth assessed using cord blood DNAm. Strengths of our work include use of prospectively collected data, large number of newborns from two birth cohorts with comparable cord blood DNAm and prenatal air pollution exposure estimates, and consideration of weekly air pollution exposure. We implemented models mutually adjusted for other exposure periods and the DLMs to account for correlations across different exposure periods and allow for investigation of critical exposure window of interest. Several limitations should be noted. Although we have weekly exposure prior to conception and through delivery with broad range of air pollution exposure and spatial variation across multiple regions of U.S, we did not explore indoor air pollution exposure or account for variations in exposure outside the home. In addition, our study should be interpreted with caution while using the epigenetic clocks for future studies. For example, the Knight clock is developed using gestational age estimated by last menstrual period in six cohorts including preterm infants, while the Bohlin clock only draws samples from a single termed infant cohort that estimated gestational age based on fetal ultrasound. In this study, we did not explore epigenetic clocks that incorporate clinical and lifestyle factors, such as plasma proteins and cigarette smoking, or extrinsic epigenetic age acceleration/deceleration, which is dependent on cell composition. Investigation of epigenetic aging along with those biologically relevant markers at various time points of pregnancy may provide additional insights on the fetal activity throughout the entire pregnancy.

In summary, exposures to O_3_, PM_2.5,_ and PM_10_ during preconception and pregnancy were associated with epigenetic age deceleration at birth. Findings of the current study contribute to the growing literature that suggests epigenetic aging is responsive to environmental factors and supports the use of epigenetic clocks as potential mediators of adverse health outcomes in related to environmental exposures.

## Data Availability

The datasets presented in this study can be found in online repositories. The names of the repository/repositories and accession number(s) can be found below: The National Institute of Mental Health Data Archive (NDA), under the collections for the EARLI study (1600) https://nda.nih.gov/edit_collection.html?id=1600 and for the MARBLES study (1946) https://nda.nih.gov/edit_collection.html?id=1946.

## Supplementary Material

Supplementary Figure S1 Box and whisker plots of ambient air pollutants. Box and whisker plots showing the distribution of **(A)** NO_2_ **(B)** O_3_ **(C)** PM_2.5_ **(D)** PM_10_ for preconception, trimester 1, trimester 2, trimester 3, and pregnancy. The boxes represent the interquartile ranges for air pollutants. The horizontal black line dividing the box represents the median.

Supplementary Figure S2 Box and whisker plots of ambient air pollutants by region. Box and whisker plots showing the distribution of **(A)** NO_2_ **(B)** O_3_ **(C)** PM_2.5_ **(D)** PM_10_ for preconception, trimester 1, trimester 2, trimester 3, and pregnancy by region. The boxes represent the interquartile ranges for air pollutants. The horizontal black line dividing the box represents the median. Two-tailed t-tests were used to compare the differences of each pollutant by region with the following symbols indicating p-value (ns: p > 0.05, ^∗^: p <= 0.05, : ^∗∗^p <= 0.01, ^∗∗∗^: p <= 0.001, ^∗∗∗∗^: p <= 0.0001).

Supplementary Figure S3 Correlation between air pollutants. Darker shades represent stronger correlations. Blue shades indicate positive correlations, while red shades indicate negative correlations, as outlined in corresponding key. Abbreviations: NO2, Nitrogen Dioxide; NOx, Nitrogen Oxides; O3, Ozone; PM2.5, Particulate Matter < 2.5 μm; PM10, Particulate Matter < 10 μm.

Supplementary Figure S4 Correlations between epigenetic age and gestational age at birth. Scatter plots showing the correlation of **(A)** epigenetic age estimated by Knight et al. **(B)** epigenetic age estimated by Bohlin et al. with the smooth line and 95% confidence interval. The correlation coefficient represents the spearman coefficient.

Supplementary Figure S5 Distributed lag models results for NO_2._ β coefficients and 95% confidence intervals from distributed lag models (DLMs) are shown for associations between **(A)** NO_2_ and epigenetic age acceleration estimated by Knight et al. **(B)** NO_2_ and epigenetic age acceleration estimated by Bohlin et al. at each week of preconception and pregnancy. All models were adjusted for child sex, maternal race/ethnicity, maternal education, year of birth, and region of the participant at recruitment. The *β* coefficient represents the difference in epigenetic age acceleration for a 10-unit difference in the pollutant. Epigenetic age acceleration was defined as the residual of epigenetic age estimated by Knight clock or Bohlin clock on gestational age at birth adjusted for cell heterogeneity.

Supplementary Figure S6 Distributed lag models results for O_3._ β coefficients and 95% confidence intervals from distributed lag models (DLMs) are shown for associations between **(A)** O_3_ and epigenetic age acceleration estimated by Knight et al. **(B)** O_3_ and epigenetic age acceleration estimated by Bohlin et al. at each week of preconception and pregnancy. All models were adjusted for child sex, maternal race/ethnicity, maternal education, year of birth, and region of the participant at recruitment. The *β* coefficient represents the difference in epigenetic age acceleration for a 10-unit difference in the pollutant. Epigenetic age acceleration was defined as the residual of epigenetic age estimated by Knight clock or Bohlin clock on gestational age at birth adjusted for cell heterogeneity.
