# Supplementary material for: Prenatal Exposure to Ambient Air Pollution and Epigenetic Aging at Birth in Newborns
Source: Front Genet. 2022 Jun 28;13:929416. doi: 10.3389/fgene.2022.929416 (PMC9274082; doi:10.3389/fgene.2022.929416)
Supplement: Supplementary file 1 [file DataSheet1.pdf]

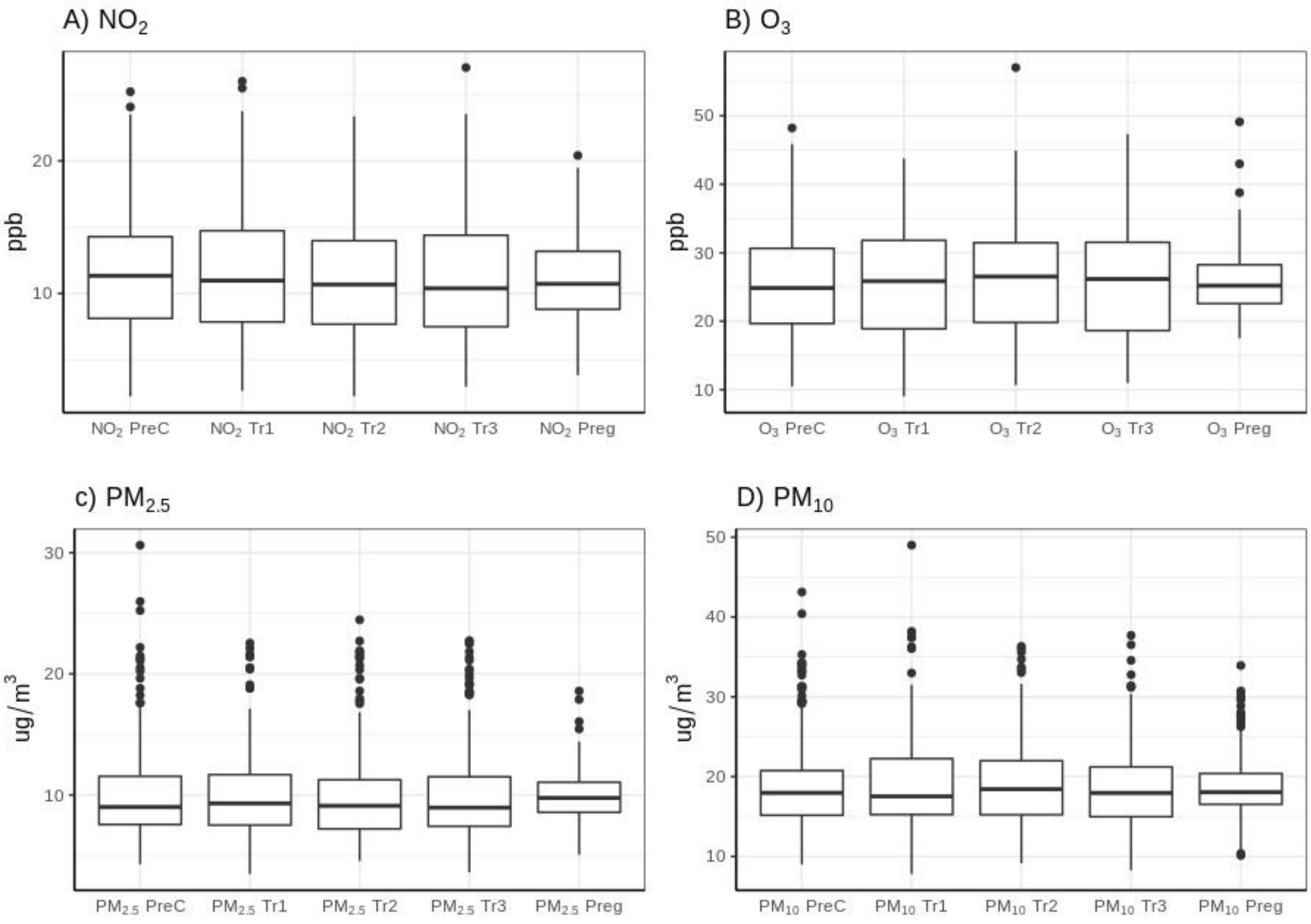

Figure S1. Box and whisker plots of ambient air pollutants. Box and whisker plots showing the distribution of A) NO_2 B) O_3 C) $PM_{2.5}$ D) PM_{10} for preconception, trimester 1, trimester 2, trimester 3, and pregnancy. The boxes represent the interquartile ranges for air pollutants. The horizontal black line dividing the box represents the median.

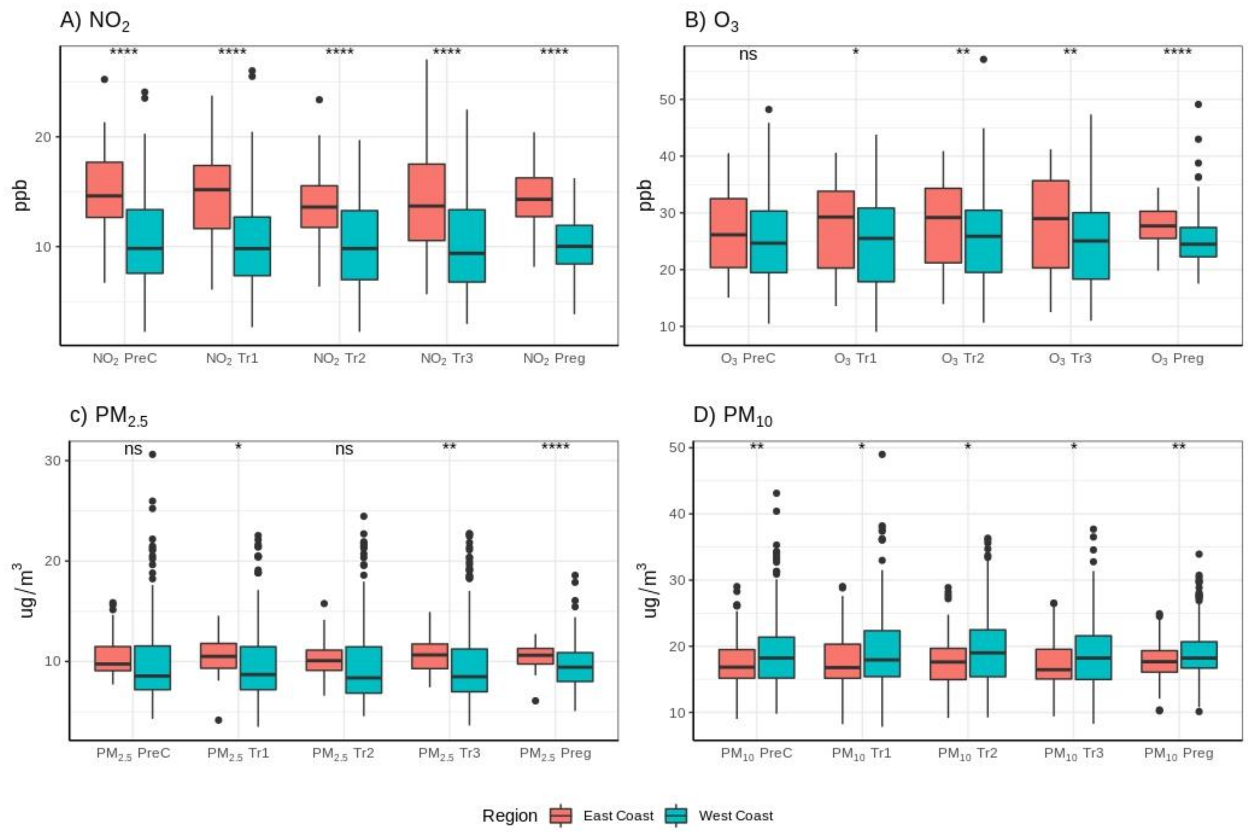

Figure S2. Box and whisker plots of ambient air pollutants by region. Box and whisker plots showing the distribution of A) NO_2 B) O_3 C) $PM_{2.5}$ D) PM_{10} for preconception, trimester 1, trimester 2, trimester 3, and pregnancy by region. The boxes represent the interquartile ranges for air pollutants. The horizontal black line dividing the box represents the median. Two-tailed t-tests were used to compare the differences of each pollutant by region with the following symbols indicating p-value (ns: p > 0.05, *: p <= 0.05, **: p <= 0.01, ***: p <= 0.001, ****: p <= 0.0001).

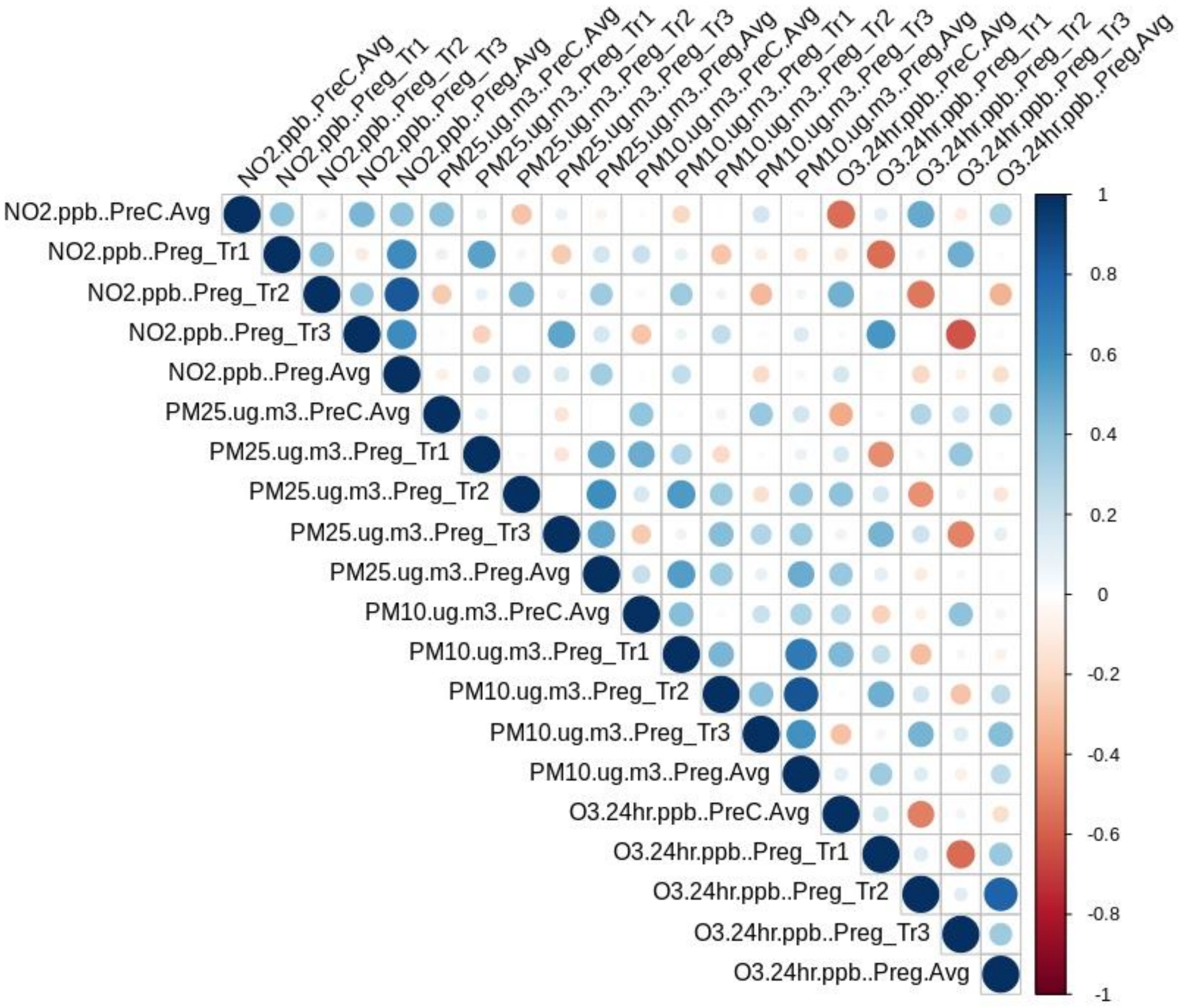

Figure S3. Correlation between air pollutants. Darker shades represent stronger correlations. Blue shades indicate positive correlations, while red shades indicate negative correlations, as outlined in corresponding key. Abbreviations: Prec.Avg: preconception average; Tr1: trimester 1; Tr2: trimester 2; Tr3: trimester 3; Preg.Avg: pregnancy average.

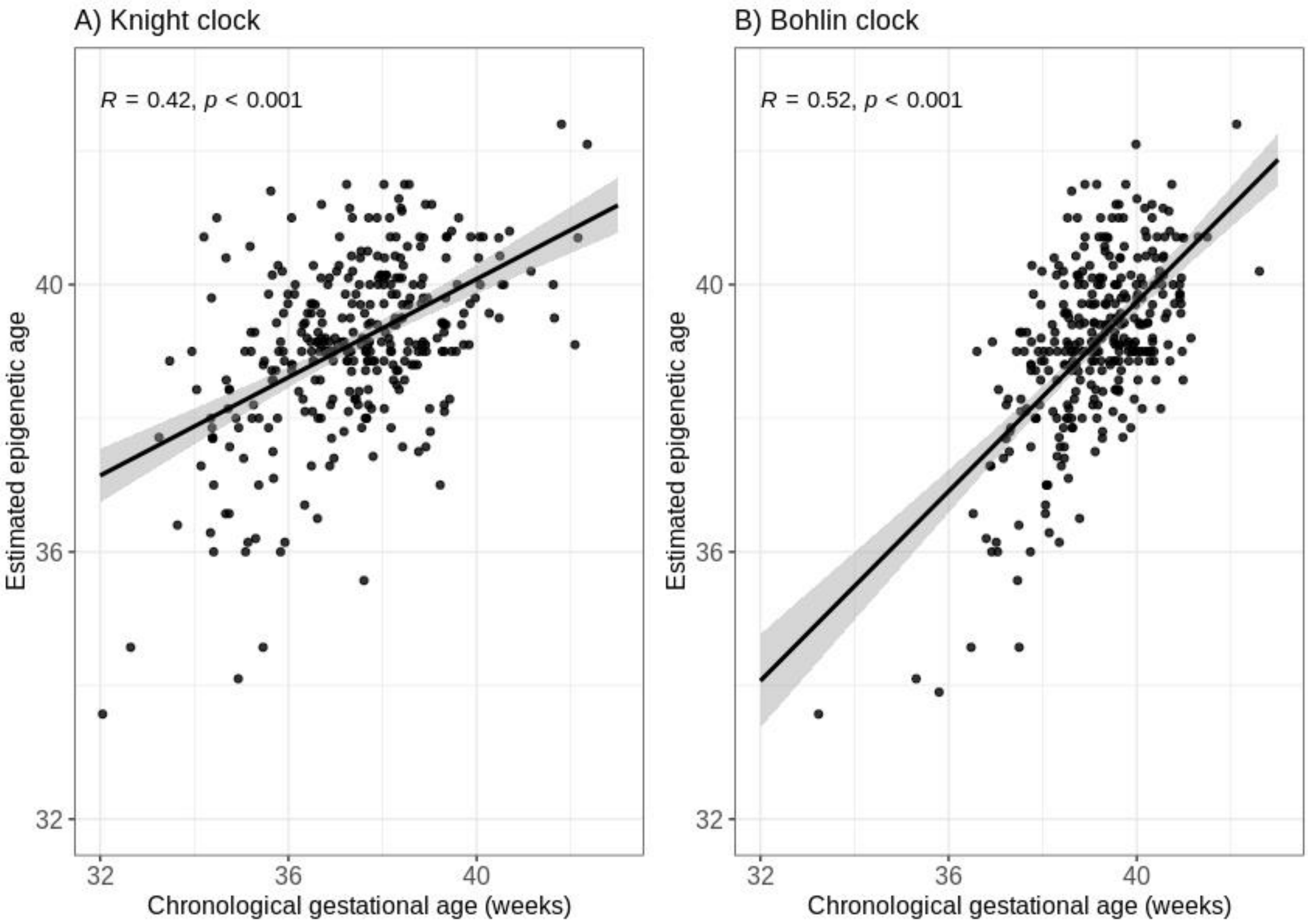

Figure S4. Correlations between epigenetic age and gestational age at birth. Scatter plots showing the correlation of A) epigenetic age estimated by Knight et al. B) epigenetic age estimated by Bohlin et al. with the smooth line and 95% confidence interval. The correlation coefficient represents the spearman coefficient.

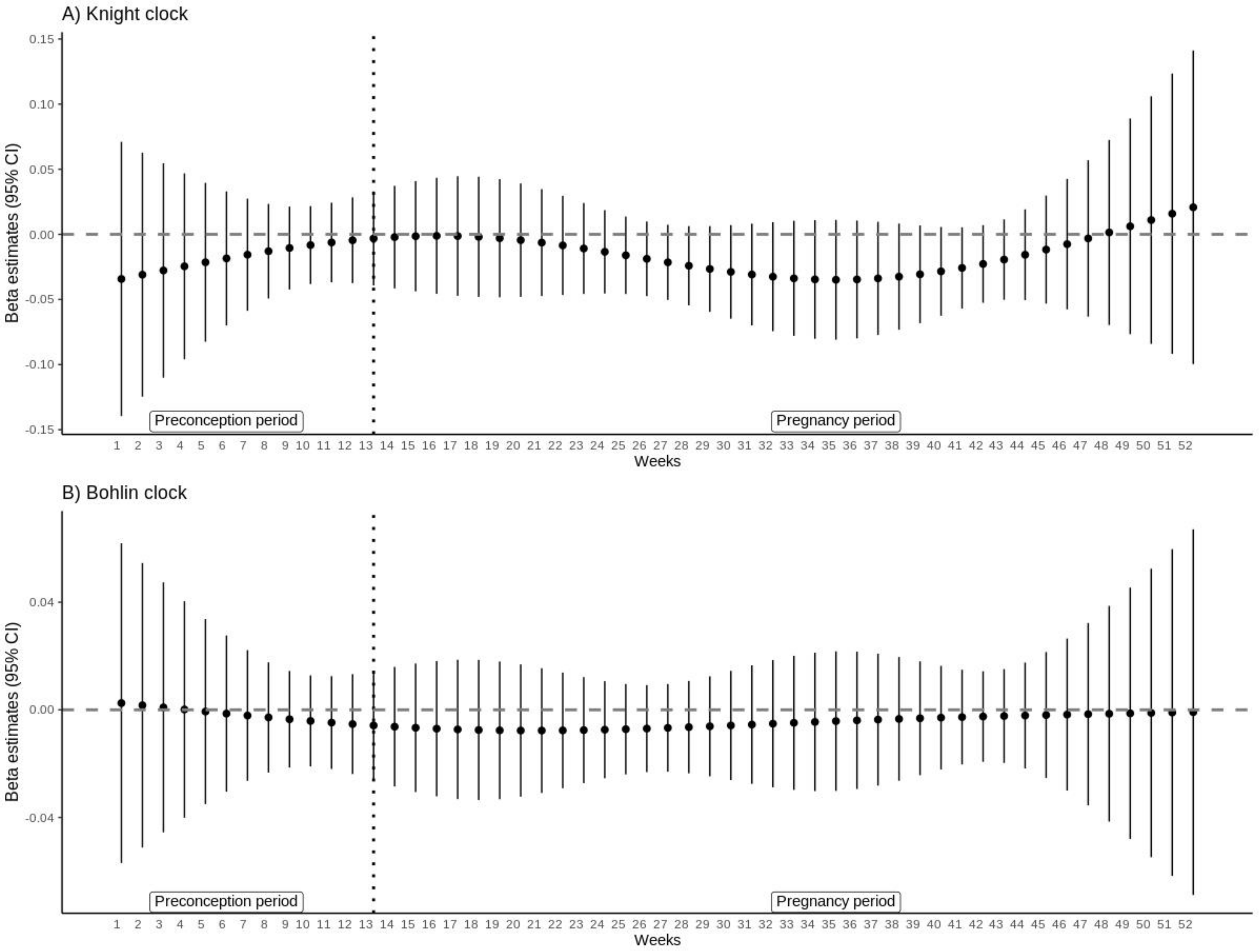

Figure S5. Distributed lag models results for NO_2. β coefficients and 95% confidence intervals from distributed lag models (DLMs) are shown for associations between A) NO_2 and epigenetic age acceleration estimated by Knight et al. B) NO_2 and epigenetic age acceleration estimated by Bohlin et al. at each week of preconception and pregnancy. All models were adjusted for child sex, maternal race/ethnicity, maternal education, year of birth, and region of the participant at recruitment. The β coefficient represents the difference in epigenetic age acceleration for a 10-unit difference in the pollutant. Epigenetic age acceleration was defined as the residual of epigenetic age estimated by Knight clock or Bohlin clock on gestational age at birth adjusted for cell heterogeneity.

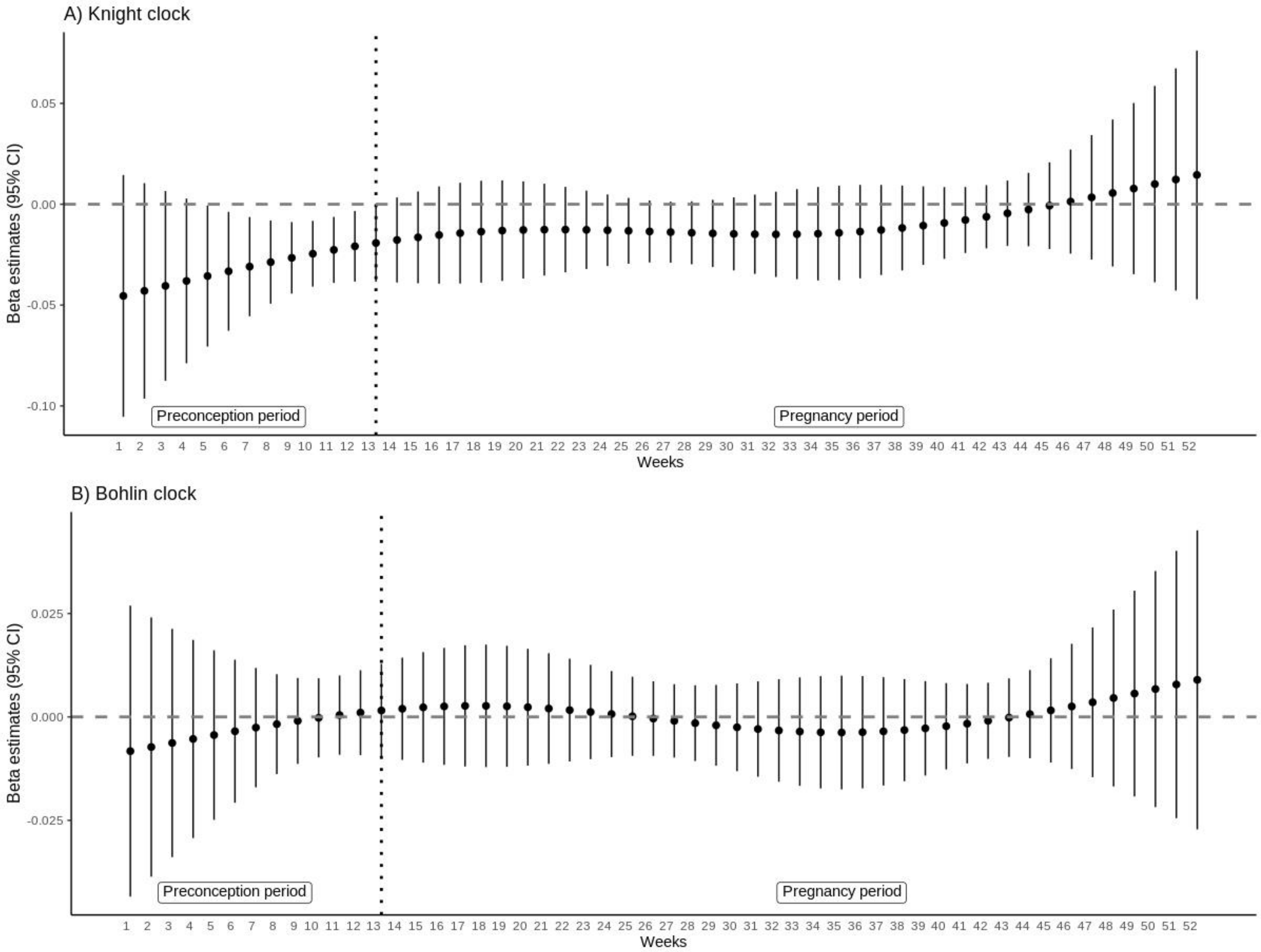

Figure S6. Distributed lag models results for O_3. β coefficients and 95% confidence intervals from distributed lag models (DLMs) are shown for associations between A) O_3 and epigenetic age acceleration estimated by Knight et al. B) O_3 and epigenetic age acceleration estimated by Bohlin et al. at each week of preconception and pregnancy All models were adjusted for child sex, maternal race/ethnicity, maternal education, year of birth, and region of the participant at recruitment. The β coefficient represents the difference in epigenetic age acceleration for a 10-unit difference in the pollutant. Epigenetic age acceleration was defined as the residual of epigenetic age estimated by Knight clock or Bohlin clock on gestational age at birth adjusted for cell heterogeneity.
